# Leveraging the Role of Dynamic Reconfigurable Antennas in Viewpoint of Industry 4.0 and Beyond

**DOI:** 10.34133/research.0110

**Published:** 2023-04-04

**Authors:** Abdul Jabbar, Muhammad Ali Jamshed, Qammer Abbasi, Muhammad Ali Imran, Masood Ur-Rehman

**Affiliations:** James Watt School of Engineering, University of Glasgow, Glasgow, UK.

## Abstract

Industry 4.0 is a digital paradigm that refers to the integration of cutting-edge computing and digital technologies into global industries because of which the state of manufacturing, communication, and control of smart industries has changed altogether. Industry 4.0 has been profoundly influenced by some major disruptive technologies such as the Internet of Things (IoT), smart sensors, machine learning and artificial intelligence, cloud computing, big data analytics, advanced robotics, augmented reality, 3D printing, and smart adaptive communication. In this review paper, we discuss physical layer-based solutions with a focus on high reliability and seamless connectivity for Industry 4.0 and beyond applications. First, we present a harmonized review of the industrial revolution journey, industrial communication infrastructure, key performance requirements, and potential sub-6-GHz frequency bands. Then, based on that, we present a comprehensive review of intelligent tunable dynamic antenna systems at sub-6 GHz as key enablers for next-generation smart industrial applications. State-of-the-art smart antenna techniques such as agile pattern reconfigurability using electrical components, machine learning- and artificial intelligence-based agile beam-scanning antennas, and beam-steerable dynamic metasurface antennas are thoroughly reviewed and emphasized. We unfolded the exciting prospects of reconfigurable dynamic antennas for intelligent and reliable connectivity in application scenarios of Industry 4.0 and beyond such as Industrial IoT and smart manufacturing.

## Introduction

The fourth industrial revolution (Industry 4.0) has evolved and embraced current technological solutions with the advancement of wireless communication technology [1,2]. The concept of smart industries and smart factories is no more surprising, and the industrial environment of major companies and businesses has been changed altogether. The industrial shift toward digitalization, automation, and cognition is a huge indication of the smart economy and societal progress. It is obvious that with the integration and potential use of key enabling technologies of 5G and beyond, worldwide smart industries can revolutionize further and soar to their full potential [3,4]. Conventionally, most of the mainstream industrial communication network is still wired, which has various impediments such as less flexibility, low mobility, installation difficulty, high maintenance cost, and restricted coverage. To mitigate such weaknesses and to serve the requirements of smart communication in modern industries, intelligent wireless communication is crucial in industrial settings. Since the antenna is the gateway of any wireless communication system and especially serves as the backbone of Internet of Things (IoT), a smart and agile antenna system can play a key role to achieve efficient modern industrial wireless communication and control [5]. In 2010, polls for the adoption of some wireless systems-related parameters were conducted to analyze the priority of various factors in industrial communication. The outcome showed the highest percentage for reliability (95.5%) as a major concern for industrial wireless communication as compared to other performance indicators, such as security, power, and ease of use [6]. In order to maintain high reliability, good coverage, high communication quality, and high throughput specifically in harsh industrial settings, an intelligent antenna system is envisaged as a key enabler [7].

**Fig. 1.**
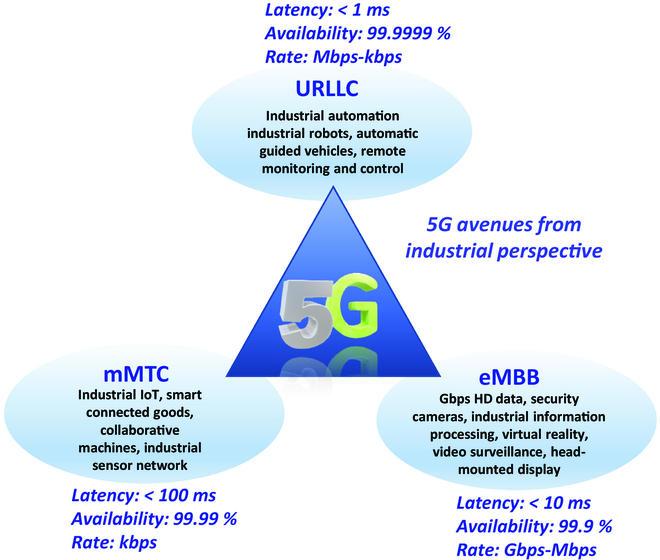
Demonstration of 5G services from an industrial point of view.

From the perspective of 5G services, the 3 main avenues are enhanced mobile broadband (eMBB), massive machine-type communications (mMTC), and ultra-reliable low-latency communication (URLLC). With a strong emphasis on machine-type communication and IoT, the industrial 5G networking technology will alter how smart factories make decisions, produce goods, and maintain operations. It will, however, coexist with Wi-Fi (Wireless Fidelity) and other wireless technologies. The application scenarios and service models of 5G under these 3 avenues are presented in different ways in the literature [8–10]. Viewing from an industrial perspective, mMTC aims to allow interaction between a substantial number of smart industrial sensors, devices, and gateways enabling autonomous environments in the industry. The eMBB aims toward high throughput for high data rate applications, such as head-mounted displays and vision-based industrial control. On the other hand, URLLC targets high reliability, security, and low latency for time-critical applications. A viewpoint of 5G from an autonomous industrial aspect is presented in Fig. 1.

In an industrial environment, the wireless infrastructure in addition to its advantages poses different challenges as well. These challenges are due to large metallic structures, the mobility of robots and vehicles, and electromagnetic interference from various electronic appliances. As a result, the reliability and performance of industrial wireless networks may be severely affected. Moreover, modern factory automation (FA) requires URLLC to accomplish mission-critical and sensitive real-time operations [11]. To demonstrate the concept of reliability in a smart factory environment, a coordinated multi-point (CoMP) transmission and reception scheme is favorable as demonstrated by Qualcomm [12]. CoMP relies on spatial multiplexing and spatial diversity techniques, i.e., transmission and reception at numerous dispersed locations with dynamic coordination between them. This helps to actively manage blockage or interference for industrial users, with a prime focus on cell-edge users.

**Fig. 2.**
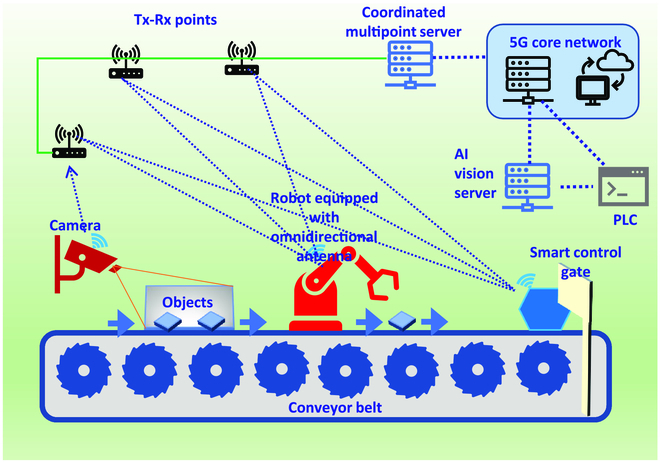
A conceptual depiction of coordinated multi-point (CoMP) connectivity for reliable industrial communication.

A conceptual demonstration of a visual-based agile CoMP system using multi-point antenna connectivity in an industrial setting (scenario of an automatic conveyor belt in FA) is shown in Fig. 2 excerpted from Ref. [12]. A camera sends visual images of the objects passing through the conveyor belt via access points (APs) to the 5G core network and then processed by an artificial intelligence (AI)-based vision server. An AI server then sends commands through a programmable logic control to the smart robotic arm to perform the task in real time. The autonomous robot picks up some objects aside as indicated by the AI server, while letting other objects pass through an automated gate. From an antenna system perspective, omnidirectional antennas (with wider beamwidth and coverage) are suitable in the CoMP network, which could connect to a new AP in case of any blockage to maintain a reliable communication link. However, in this concept, several coordinated transmit antennas or APs are involved, which might not be cost-effective and can produce excessive signal processing overhead at the AI server.

It does not need to be mentioned that the antenna design research has been widely conducted in sub-6-GHz industrial, scientific, and medical (ISM) bands, such as 2.4 and 5 GHz, for a plethora of applications. However, it is important to focus on intelligent dynamic reconfigurable antenna systems that offer real-time reconfigurable radiation beams and agility to cater for varying industrial environments. Dynamic metasurface antennas (DMAs), agile beam steerable conventional antennas, and machine learning (ML)- and AI-based reconfigurable antennas are the key physical layer (PHY)-based antenna solutions for next-generation industrial applications, which are the focus of this review paper. Moreover, millimeter-wave (mmWave) spectra such as the 60-GHz ISM band and mmWave antenna designs have unfolded many opportunities for smart industrial applications [13–18]. However, the scope of this review paper mainly includes sub-6-GHz smart antenna designs for future industrial applications and FA scenarios. This is because the sub-6-GHz band has been pervasive and predominantly existing in mainstream industrial environments, the channel behaviors are well known, and the hardware implementation does not pose severe challenges.

A number of surveys and reviews are available in the literature covering different aspects of industrial wireless communication such as mmWave industrial communication [16], industrial wireless communication protocols, smart manufacturing and Industrial IoT [19,20], and similarly many other aspects of Industry 4.0 [17,21–28]. To understand the requirements of industrial wireless communication from the viewpoint of Industry 4.0 and beyond, a combined study of modern industrial wireless infrastructure along with the desired agile antenna systems for industrial applications and seamless connectivity is necessary. However, it is explored that from the perspective of Industry 4.0 and beyond applications, a thorough review of state-of-the-art smart reconfigurable antenna systems in sub-6-GHz bands as PHY solutions is lacking. To fill this gap, we present a comprehensive review of state-of-the-art dynamic reconfigurable antenna designs at sub-6-GHz bands, which are suitable for next-generation industrial applications.

This review paper is organized as follows. In the Industrial Revolution Towards Industry 4.0 and Beyond section, we shed light on industrial transformation toward Industry 4.0 and beyond, along with key digital enablers of Industry 4.0. In the Industrial Activity Categorization and Key Performance Indicators section, we present PHY requirements and key performance indicators (KPIs) of industrial use cases. In the Industrial Wireless Standards, Infrastructure, and Potential Sub-6 GHz Frequency Bands section, we review potential sub-6-GHz frequency bands for wireless industrial communication, suitable for smart antenna designs. Next, in the Review of Sub-6 GHz Intelligent Antenna Designs section, we present a comprehensive review of smart reconfigurable antenna systems for Industry 4.0 and beyond applications. Some critical antenna design challenges are highlighted in the Critical Smart Antenna Design Challenges section. Finally, we conclude this review with our perspectives on future developments and challenges in this subfield.

## Industrial Revolution Toward Industry 4.0 and Beyond

**Fig. 3.**
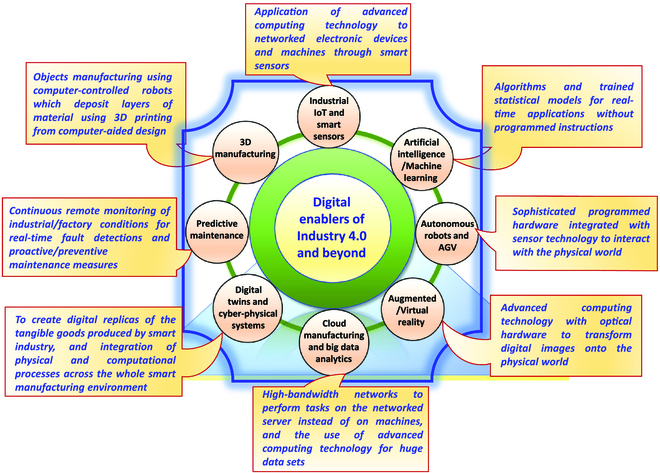
Key digital drivers of Industry 4.0 and beyond.

Since the dawn of the industrial age, technical advancements have led to paradigm shifts known as industrial revolutions. At present, Industry 4.0 is being envisaged, which is mainly about the substantial transformation happening in the way goods are produced and delivered, thus shifting toward flexible factory and industrial automation [3]. Advanced smart wireless communication, AI, Industrial IoT, digital twins, cyber-physical systems (CPS), and augmented reality are considered to be the key players for smart industries under the ambit of Industry 4.0 [4,29,30]. Advanced wireless technology with real-time reconfigurability is indispensable for URLLC, security, and robust connectivity in smart industries. As a result, a smart antenna system is envisioned as the backbone for dynamic industrial communication, ensuring adaptable and flexible industrial processes [2,31,32]. Some of the key digital drivers of Industry 4.0 excerpted from Ref. [33] are shown in Fig. 3.

**Fig. 4.**
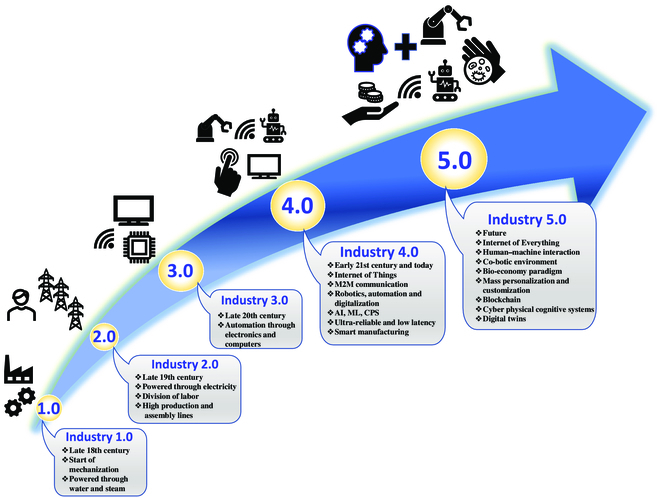
A demonstration of the industrial transformations and futuristic trends.

The industrial revolution has come a long way to see the digitalized sophisticated era. Since the focus of this paper is on the role of smart beam-scanning antennas for Industry 4.0 and beyond, the discussion on the whole industrial revolution process and its other avenues is beyond the scope of this work. An industrial transformational journey is demonstrated in Fig. 4. For the sake of completion, we shed some light on Industry 5.0 and 6.0. It is worth mentioning here that Industry 4.0 is not the apex of the modern smart industrial world, rather the visualization and conceptualization of Industry 5.0 are under anticipation in research. This is due to the fast-growing digital technologies, AI-based solutions, and human inclusion to promote collaborative work. Industry 5.0 emphasizes the bio-economy paradigm that brings together biotechnology, science, society, and economy for a sustainable smart industrial environment in collaboration with human intelligence and AI [34–38]. Its goal is to leverage the ingenuity of human experts in collaboration with machines and encourages a bio-economic paradigm [39,40]. Yet, to the surprise of readers, this is not the end and researchers have put forward the notion of Industry 6.0 [41], the details of which are, however, beyond the scope of this paper. Nevertheless, Industry 4.0 is still undergoing practical realization and has not yet been implemented pervasively (as industrial evolution is a slow process). Accordingly, in this paper, we safely refer to these so-called industrial generations as “Industry 4.0 and beyond”.

## Industrial Activity Categorization and Key Performance Indicators

The industrial automation process is fundamentally divided into 2 main categories, viz., FA and process automation (PA) [6,42]. FA refers to the production of goods and is considered a discrete automation process. It generates bursty data as opposed to streaming data and poses stringent latency requirements of up to a few milliseconds along with a shorter communication range (latency from 1 to 50 ms and distance up to tens of meters) [1,43]. The performance metrics such as reliability, low latency, and real-time capability are given more priority in FA, with less stringent requirements on power consumption, which is due to the nature of FA operations.

PA refers to batch processing and involves the monitoring and control of operations, like heating, stirring, pumping, and cooling as well as control of fluids such as oil, gas, and water. The PA applications transmit data in a streamed manner at regular intervals and latency requirements are less stringent (usually >100 ms) and the communication range is about 100 to 500 m. Low-frequency point-to-multipoint antennas can efficiently serve PA. However, power consumption is crucial in PA applications. Furthermore, power system automation and power electronics control are vital demanding scenarios for modern industrial operations [44]. The former is concerned with the control of power distribution in the industrial environment, whereas the latter deals with the synchronized control of power electronics devices.

**Fig. 5.**
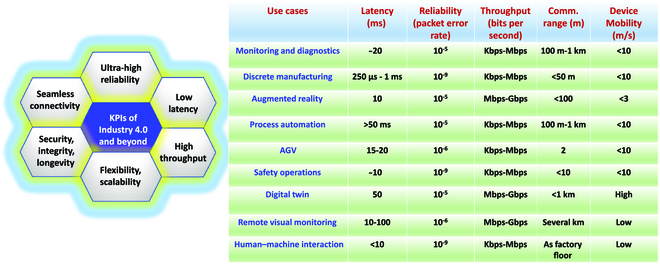
KPIs of Industry 4.0 and beyond along with smart manufacturing use cases and PHY wireless communication requirements.

The required quality of service and quality of data in terms of low latency, high reliability, high throughput, seamless connectivity, and agility are crucial for FA and other industrial applications such as smart manufacturing [45–47]. Some high-level use cases of smart manufacturing such as digital twins, CPS, predictive maintenance, remote visual monitoring and control, remote surveillance and fault detection, intelligent logistics, human–machine interaction, and collaborative machines are highlighted in Refs. [20,48]. With CPS, the physical processes are constantly analyzed and managed in the digital paradigm via a computational process that has a direct impact on real-world manufacturing activities. The feedback from physical processes impacts computational methods in an optimization feedback loop. Moreover, with the use of digital twins, smart manufacturing could utilize digital replicas of the tangible goods produced in an industrial environment [29]. Industrial IoT is a key driver associated with digital twins to maximize production efficiency by foreseeing production-related issues. Such failures may thus be prevented before they affect manufacturing, decreasing plant downtime and related revenue losses [5,20]. The notion of fully automated manufacturing industries based on collaborative machines and machine-to-machine communication gained traction with the inception of Industry 4.0. However, as stated in the Introduction, collaborative human–machine interaction is emphasized under the ambit of Industry 5.0 to enhance mass personalization and the bio-economy paradigm [5,34]. The KPIs and performance requirements of some major use cases of smart manufacturing under the ambit of Industry 4.0 excerpted from Refs. [2,20,44] and Ref. [48] are presented in Fig. 5.

To achieve high communication performance, the PHY-based solutions, for instance, smart antenna systems with aided features such as dynamic reconfigurability, beamforming array, and multiple-input–multiple-output (MIMO), can enhance the spectral efficiency and throughput of industrial communication [31,32,49,50]. At sub-6 GHz, the communication channel, wave propagation conditions, and hardware capabilities are well studied, and path losses are well managed by a single antenna or a few antennas. To avoid sub-6-GHz spectral congestion and mitigate spectral interference, smart reconfigurable antenna techniques such as frequency reconfigurability, polarization reconfigurability, and especially radiation pattern reconfigurability can be employed to unleash the potential of flexible and smart industrial manufacturing and other applications [24,51–54].

## Industrial Wireless Standards, Infrastructure, and Potential Sub-6-GHz Frequency Bands

Modern industrial manufacturing increasingly depends on wireless connectivity and efficient wireless industrial automation systems are necessary for Industry 4.0 and smart manufacturing [2,20]. The usage of networked machinery, sensors, and actuators within modernized factories leads to the notion of Industrial IoT. For these IoT implementations, wireless system adoption is of paramount importance. The cost of wired networks and capital such as deployment of cables, networking hardware, and installation manpower is greatly reduced by wireless automation. Wireless requirements must be defined to realize the advantages of wireless communication systems inside such industries and to enable the deployment of wireless systems at the factory-floor level [44]. Therefore, to design intelligent antenna systems for next-generation wireless industrial applications, the knowledge of potential operating frequency bands is crucial. Various wireless communication protocols exist for industrial applications such as IEEE 802.11 (Wi-Fi family) for wireless local area networks (WLAN), IEEE 802.15.1 for wireless personal area networks (WPAN), and IEEE 802.15.4 for low data-rate private area networks (PAN) [6,20]. Furthermore, the prevalent industrial wireless standards are Wireless HART (Highway Addressable Remote Transducer), ISA100.11a (International Society of Automation), WIA-PA (Wireless networks for Industrial Automation–Process Automation), WIA-FA (Wireless networks for Industrial Automation–Factory Automation), WSAN-FA (Wireless Sensor Actuator Network for Factory Automation), WISA (Wireless Interface for Sensors and Actuators), Wi-Fi, Bluetooth, Zigbee, and LoRa (Long Range) [6]. These standards mainly operate in license-free sub-6-GHz ISM bands, such as 2.4 and 5 GHz. An overview of the adoption of industrial wireless standards, their key features, and their similarities and differences are studied in Refs. [6,20] and Ref. [55]. Table 1 provides a summary of the existing sub-6-GHz wireless protocols that may be employed for a variety of applications in Industry 4.0 and beyond.

**Table 1.** Existing industrial wireless communication standards at sub-6 and sub-7 GHz.

Wireless standard	IEEE PHY standard	Operatingfrequency band(GHz)
WirelessHART	802.15.4	2.4
ISA100; WIA-PA	802.15.4	2.4
Zigbee	802.15.4	2.4
WISA/WSAN-FA	802.15.1	2.4
Bluetooth	802.15.1	2.4
WIA-FA	802.11	2.4
Wi-Fi 5	802.11ac	5
Wi-Fi 6	802.11ax	2.4/5.15–5.83
Wi-Fi 6E	802.11ax	2.4/5.92–7.125
Wi-Fi 7	802.11be	2.4/5.92–7.125

**Fig. 6.**
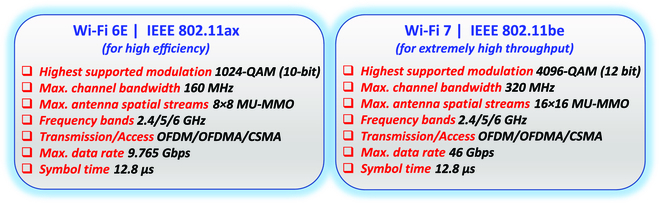
A summary of some key physical layer parameters for Wi-Fi 6 and Wi-Fi 7.

The sub-6-GHz band is spectrally congested and suffers from various impediments such as spectral interference and low available bandwidth. Industry 4.0 would demand high data rates and high-quality connectivity for various smart applications, such as time-sensitive networking (TSN), intelligent logistics, remote visual monitoring, and automatic guided vehicles (AGVs). For these applications, conventional sub-6-GHz bands show high latency and spectral congestion. As a result, the KPIs of Industry 4.0 and beyond may be compromised severely. In order to mitigate these issues and to achieve high throughput as well as high bandwidth for modern industrial operations, Wi-Fi-6/6E [56–59] and Wi-Fi-7 [60–65] have been proposed to boost the network performance at lower-frequency bands. Wi-Fi 6E and Wi-Fi 7 standards span beyond 6 GHz and are sometimes referred to as sub-7-GHz bands as well. Any new generation of radio technology always tries to take advantage of the use of new spectrum bands as they become available because the spectrum is analogous to the air that wireless networks breathe. Therefore, the new generation of Wi-Fi accepts the use of the 6-GHz spectrum as an immediate method to boost peak throughput in 802.11be (Wi-Fi 7) and high network efficiency in 802.11ax (Wi-Fi 6). A summary of some key PHY parameters of Wi-Fi 6 and Wi-Fi 7 is presented in Fig. 6.

### Wi-Fi 6/6E

IEEE 802.11ax standard (Wi-Fi 6 and 6E) is designed where greater bandwidth, low latency, high throughput, and excellent network efficiency are all important considerations and are well suited for industrial applications. Wi-Fi 6/6E provides functionalities that are implemented in industrial-grade components. The Wi-Fi 6/6E is backed by OFDMA (orthogonal frequency-division multiple access), which represents an important advancement in Wi-Fi 6's ability to provide effective communication [56]. Using OFDMA, the communication channel is split up into several resource units, or smaller channels. Different industrial entities may use these subchannels in a variety of bundles. This enables simultaneous data transmission, which reduces the time between packets. Furthermore, as Wi-Fi 6/6E can serve more users in less time, it can achieve substantially reduced latency as well as a more equitable distribution of bandwidth among clients.

Another aspect is that since only a small portion of the channel must be used for each transmission, it can offer a more effective transmission of small data packets. This prevents bandwidth from being wasted [57,58]. For instance, this enables various industrial entities, such as massive industrial sensor network deployment, AGVs, and conveyor belts, to react to unforeseen situations. Consequently, it can be operated at a faster speed without increasing risk. This also lays the groundwork for addressing in parallel the increasingly common demands for dependable real time in automation components and data-intensive applications like the transmission of high-definition images and videos for vision-based TSN or remote visual monitoring in FA [56,65]. Additional features included in the Wi-Fi 6 standard are the 8 × 8 multi-user (MU) MIMO and a higher modulation scheme such as 1,024-Quadrature Amplitude Modulation (QAM), which both increase the nominal data rate and enhance overall industrial WLAN performance [56].

### Wi-Fi 7

The IEEE 802.11be standard, also marketed as Wi-Fi 7 or Extremely High Throughput (EHT) [66,67], is a new addition to the Wi-Fi standard that aims to increase the nominal throughput to as much as 46 Gbps through spatial multiplexing and spatial diversity [60,62]. The PHY layer is improved via double bandwidth to 320 MHz, an increase in modulation to 4,096-QAM, and an increase in the number of spatial streams to 16 × 16 MU-MIMO [63,65]. To achieve exceptionally high throughput, the EHT physical layer provides higher bandwidth, more spatial streams, and a higher modulation method. For a single PHY link, the maximum throughput can be calculated by using the formula below [68]:

$$\text{Max}.\text{PHY data rate}=\frac{N_{\text{CBPS}}\times N_{\mathrm{SD}}\times N_{\mathrm{SS}}\times R}{T_{\mathrm{SYM}}}$$

where *N_CBPS_* is the number of coded bits per OFDM symbol (12 bits for 4,096-QAM)), *N_SD_* is the number of data subcarriers depending on channel bandwidth (3,920 for the 320-MHz channel), *N_SS_* is the number of spatial data streams generated by the antenna (16 for 16 × 16 MIMO), *R* is the code rate (5/6 for 4,096-QAM), and *T_SYM_* is the OFDM symbol duration (12.8 μs) including guard time. For a typical theoretical calculation of the maximum PHY data rate achieved via the Wi-Fi 7 link, consider a link using 4,096-QAM modulation (*N_CPBS_* = 12 bits), a 320-MHz channel (*N_SD_* = 3,920), a 16 × 16 MIMO (*N_SS_* = 16), a code rate of 5/6 (*R* = 5/6), and a short guard interval time of 0.8 μs (*T_SYM_* = 12.8 + 0.8); the maximum achievable link throughput is 49 Gbps.

Apart from Standard Ethernet (IEEE 802.3) and 5G technologies, the potential solution for next-generation industrial applications is anticipated to be Wi-Fi 6/6E (IEEE 802.11ax) and Wi-Fi 7 (IEEE 802.11be). The technologies will likely be integrated with advancements for time-sensitive communication, such as the TSN standards outlined by IEEE 802.1, to create a solution in an Industry 4.0 context. Additionally, to ensure fair coexistence with independent APs deployed in the same coverage area and to optimize channel access, the implementation of Distributed-MIMO in 802.11be would necessitate the design of new distributed carrier sense multiple access with collision avoidance mechanisms that are compliant with regulations [67].

Based on the foundation set above (with the outlook of industrial wireless infrastructure, network performance requirements, and existing frequency bands), real-time intelligent beam-steering antennas achieved by different techniques [69–73] are envisaged as potential enablers for Industry 4.0 and beyond applications. It is imperative to design agile beam-steering antennas for industrial needs, and in the next section, we present a comprehensive review of state-of-the-art dynamic reconfigurable antennas.

## Review of Sub-6-GHz Intelligent Antenna Designs

A plethora of antenna designs is presented in literature at sub-6-GHz bands, typically at ISM bands (2.4 and 5 GHz). Recently, antenna designs have been reported for Wi-Fi 6/6E/7 bands [74–77], as these are efficient frequency bands for modern industrial applications. Usually, at the sub-6-GHz ISM band, single-element antennas are designed for wireless communication, without dynamic controllable features. However, a single-element antenna design without any controllable features (such as beam-steering, frequency reconfigurability, pattern agility, or polarization control) cannot steer the beams. Such antenna designs cannot mitigate spectral congestion, interference, and jamming issues in a dynamic industrial environment. When smart features such as the real-time capability to steer radiation beam, adaptive beamforming and dynamic radiation pattern reconfigurability, or frequency and polarization agility are employed with antenna designs, such a system can be called a smart antenna system [78–81]. Reconfigurable directional antennas that transmit and receive power in a specified direction, as opposed to isotropic emission of omnidirectional antennas, offer a promising way to mitigate non-line-of-sight issues, and avoid collisions. As a result, 2 improvements are achieved, i.e., a higher antenna gain, which, in some cases, can increase communication range and cut down on the number of nodes needed to cover an area, and a lower transmit power requirement [49].

**Fig. 7.**
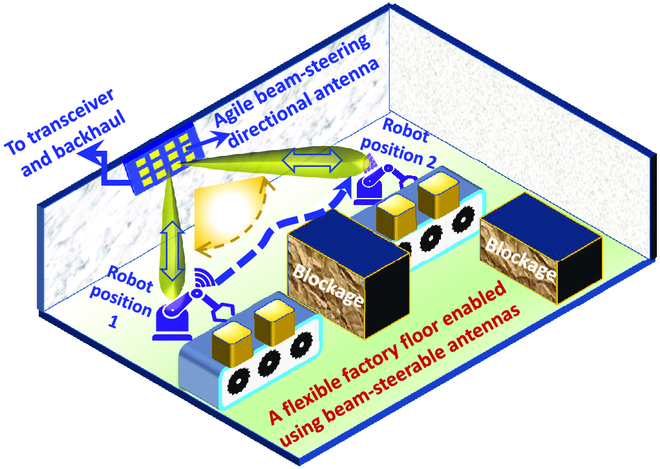
A high-level conceptual illustration of an agile beam-steering antenna covering the dynamic movement of AGV/autonomous robot enabling reconfigurable and flexible wireless communication on a smart factory floor.

The industrial shift from wired to wireless communication has made smart and flexible manufacturing possible. A smart manufacturing industrial environment is expected to be flexible or capable of reconfiguring itself based on flexible production demands (unlike in the case of fixed wired networks). In such a dynamic factory scenario, the APs are usually fixed, while the industrial entities such as autonomous robots or AGVs are moving. Using a single smart antenna with beam-steerability feature at the AP end (depending on its beam-scanning range), real-time seamless connectivity can be maintained while reducing the requirement of various APs (as opposed to the CoMP case presented in the Introduction, where multiple APs are required for reliability). Thus, an agile beam-steering (or beam-scanning) antenna with a wide beam-scanning range is anticipated as a potential PHY-based candidate to envisage URLLC in next-generation industrial applications. Moreover, a single beam-steerable antenna with a wide scanning range would be more cost-effective than utilizing numerous APs to provide continuous connectivity. Note that the terms beam-steering, beam-scanning, and beam-switching are interchangeably used in this paper. A conceptual depiction of an agile beam-steerable antenna to support reconfigurable industrial applications such as flexible manufacturing is shown in Fig. 7.

Different types of reconfigurable antennas are designed based on the required applications. Polarization reconfigurability helps in case of antenna misalignment and to recover losses due to polarization mismatches. Frequency reconfigurability is beneficial to switch the operating frequency band while using the same antenna (such as from the 2.4-GHz to the 5-GHz band and vice versa). However, radiation pattern reconfigurability is the most desirable in varying FA and industrial conditions to ensure URLLC and seamless connectivity.

The smart antenna can be realized using reconfigurable techniques that can be preconfigured to achieve diversity gain and coverage sectors. It can also come up with an adaptive beamforming capability that steers the nulls to the interference, and radiated beam toward the targeted client. Therefore, to obtain high gain and smart performance for modern industrial communication, the array designs with beamforming, reconfigurability, and real-time controllability features are obvious preferences [7,51,52,82,83]. Reconfigurability can be categorized into electrical, optical, mechanical, and smart materials-based techniques. The electrical reconfigurability in smart antennas is achieved by various methods such as positive-intrinsic-negative (PIN) diodes, varactors, and radio-frequency (RF) micro-electromechanical system (MEMS) switches with different performance values [84]. Detailed knowledge about the reconfigurability techniques in the antenna design is presented in Refs. [24,54,84,85].

### Reconfigurable conventional smart antennas

Beam-steering antennas are enablers to reduce data collision of multimode communication, avoid blockages, and provide seamless connectivity in dynamic industrial applications, such as Industrial IoT and dynamic reconfigurable connectivity [49,50]. By directing the radiation beam in a certain direction to achieve more stable data transmission, the antenna system with beam-scanning capabilities can filter the interference of other channels. In addition, the beam-scanning antenna may optimize the spatial power allocation based on the amount of communication data of the node, which offers the chance to lower the data collision rate, average energy, and packet transmission delay [32].

**Fig. 8.**
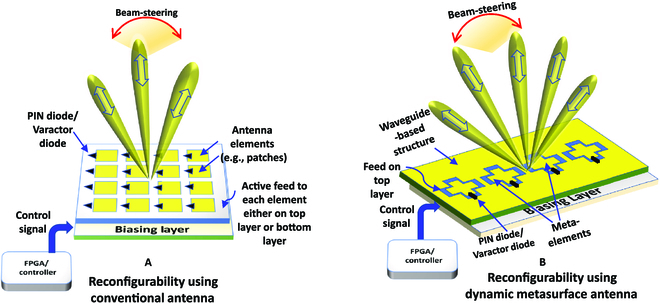
(A) Demonstration of a pattern agile reconfigurable antenna based on conventional antenna elements. PIN diode with each radiating element is responsible to change the phase in antenna elements controlled by external control circuitry, such as FPGA. Through different switching sequences, beam-steering is achieved. (B) Demonstration of a tunable DMA. Beam-scanning is controlled by the coding sequence of PIN diodes controlling the phase state of each meta-element.

The concept of beam-steering using varactor diodes or PIN diodes loaded with conventional antenna elements (such as patch elements) is usually employed. Varactor diodes provide continuous beam-scanning, whereas PIN diodes provide discrete beam-steering. By switching the state of PIN diodes loaded with each element (either “state 1” for forward biased, or “state 0” for reverse biased), the direction of current polarization on elements can be changed to modify the radiation phase. Consequently, a phase difference of 180° between 2 states can be achieved in the antenna element to mimic so-called digital coding, and the antenna element can be called a “1-bit digital element”. Similarly, if 4 different phase states are produced in a single-element antenna using multiple PIN diodes, it is referred to as “2-bit” phase control. By encoding different coding combinations of the so-called digital elements in an antenna array, various types of electromagnetic beams can be produced and controlled [53]. Real-time dynamic control can be achieved by using a microcontroller unit or field programmable gate array (FPGA) to create a real-time link between antenna elements and an external control unit [53,69,78]. A generalized conceptual representation of this concept is shown in Fig. 8A.

It is noteworthy that *n-bit* phase sequence can be generated by integrating multiple PIN diodes (or varactor diodes) in specific arrangements either directly with radiating elements, or with the feed network. In Ref. [52], a polarization and frequency agile antenna with digital-controllable radiation patterns using a field-programmable coding method is presented. This antenna array can steer the beam to 4 different radiation forms and achieve 3 different linear polarization states, which provide a suitable solution for anti-jamming or blocked industrial communication. A 360° beam-steering patch antenna array with parasitic elements is presented in Ref. [86]. In Ref. [78], a 4 × 4 digitally controllable planar patch array antenna is designed for pattern agility. The switching state of each element represents the digital code 0 (OFF state) and 1 (ON state). This coding sequence can be independently controlled, and 13 different radiation beams of the array antenna can be dynamically controlled through different coding sequences using a microcontroller. In Ref. [51], a multifunctional 1×4 phased array patch antenna is reported with wideband frequency agility and simultaneous polarization reconfiguration. A polarization control feed network is proposed in Ref. [51], which consists of RF switches, a power divider, and a coupled line phase shifter for smart antenna control.

### Reconfigurable DMAs

In recent years, metamaterial-based antennas have gained huge attention because of their excellent electromagnetic performance and advantages for improving antenna performance [87–93]. Interestingly, the concept of digital coding (as discussed above) was first suggested in metasurfaces [94], and later on, its analog effect was employed in conventional antenna elements as well. It has been demonstrated in the literature that metasurfaces and metasurface-based antennas can manipulate wavefronts of anomalous reflection and refraction waves, manage wave polarization, and perform computational imaging systems [70,94,95]. The developments in coded-aperture-based metasurfaces can dynamically change functions through programmability, thus creating a connection between the virtual and real worlds. Metasurface apertures, in particular, have been created as a type of holographic antenna in which metamaterial elements are used to form a hologram excited by a reference feed wave [70,71,73,89,96–99]. Contrary to large transmit or reflect array antennas that use sparse feed (prone to occlusion), the DMAs are directly circuit fed from the side and have the advantages of low profile and easy integration with the microwave and mmWave circuits, due to which they become a suitable candidate for industrial applications and Industrial IoT [32]. These are the next-generation antennas with striking applications in dynamic beam-steering, massive MIMO [100,101], sensing, localization, and multi-object tracking [102].

In DMAs, often a complementary electric inductive-capacitive (cELC) meta-element is employed as a basic radiator [103]. A number of such cELC meta-elements are integrated with PIN diodes (or varactor diodes) for desired phase switching and subsequent beam-steering, as depicted in Fig. 8B. It is instructive to mention that as opposed to the ON and OFF state working conditions for PIN diodes with conventional patch antennas, the cELC elements are short-circuited when the diodes are in the forward-biased state (ON state). As a result, the meta-elements are weakly radiating. On the other hand, the electrical properties of the cELC elements are preserved by the high impedance of PIN diode when it is reverse-biased (OFF or open-circuited), and meta-elements intensely radiate. In case of conventional antenna type (such as patch antenna array), the individual antenna element is always in radiating state as it directly gets the feed from RF source. The state of the switching element (PIN diode or varactor diode) is used to change the current distribution (phase) on each antenna element. Consequently, the resultant radiation pattern or polarization alters.

Waveguide-based or substrate-integrated waveguide (SIW)-based radiative metasurfaces have been proposed in the literature because of the benefits of the SIW such as low cost, low leakage loss, suitability for high frequency, and natural compatibility with print circuits [89,103–106]. It is important to mention here that an array (or MIMO array) of DMA can also be designed by repeating 1-D microstrip arrangement into planar topology with more feed ports (or power division), such as demonstrated in Ref. [50]. This can lead to beam-steering in both azimuth and elevation planes (known as 2-D beamforming/beam-scanning). An electromagnetic-compliant narrowband communication model for a generic DMA-based system is presented in Ref. [107].

Waveguide-fed metasurfaces excite metamaterial radiators in the form of conducting walls using a waveguide mode. The incident field then induces a resonance in each element, allowing energy to leak out of the waveguide selectively. The net radiation pattern of the aperture is then constructed by superimposing the radiation from each meta-element [90,93]. A programmable metasurface antenna may generate multibeam or directional beams with changeable beamwidth and beam directions by manipulating various digital codes, and dynamic radiation patterns can be achieved. Furthermore, the design guidelines and analysis of DMAs can be found in Refs. [103,104,108]. It is noteworthy here that some DMAs reviewed in this work may be at different frequency bands than 2.4 GHz, 5 GHz, or Wi-Fi 6/7 ISM bands; however, a similar design approach can be employed at the required ISM bands for industrial applications to achieve real-time beam reconfigurability by mere dimensional scalability.

Finally, a remarkable application of DMAs is looming in Industrial IoT [19,32,50]. The Industrial IoT is a subset of IoT that combines machine-to-machine and industrial communication technology with automation applications. It opens the door to a more in-depth understanding of the industrial process, enabling more efficient and sustainable production. Nonetheless, efficient wireless communication is critical to meeting the flexibility and scalability requirements of Industrial IoT connectivity. Typically, Industrial IoT applications demand minimal throughput per node, with the capacity not being a key barrier. Instead, low latency, energy efficiency, affordability, dependability, and security/privacy are more desirable characteristics for connecting a large number of devices to the internet at a low cost, despite restricted hardware capabilities and energy resources (e.g., small batteries) [19].

**Fig. 9.**
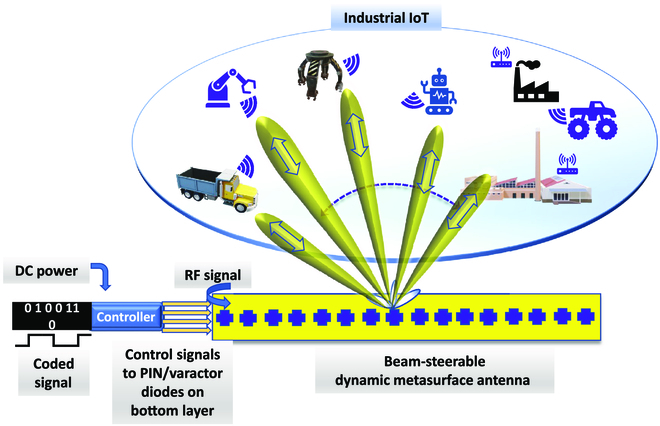
A conceptual depiction of the application of a beam-steerable DMA for Industrial IoT applications.

In Industrial IoT, a large number of communication links, especially at a relay communication node like a central base station, may cause data packet collisions and lower the network's data transmission efficiency. To solve this problem, a beam-steering antenna system can be utilized, which can transmit data more steadily by steering the radiation pattern to block interference from adjacent channels. Additionally, a beam-steering antenna may optimize the spatial power allocation and lower the data collision rate, average energy, and packet transmission delay [49], thus paving the way to envisage efficient Industrial IoT. It has been presented in Ref. [49] that compared with solutions based on omnidirectional antennas, nodes equipped with dynamic beam-steering antennas allow 88% lower energy consumption and 24% lower data collision. For seamless communication in Industrial IoT applications, DMAs with beam-steering capabilities can be deployed to lower the hardware cost of placing large-scale transmit antennas at the transmitting nodes (base stations). A conceptual realization of this concept is shown in Fig. 9.

### AI- and ML-based smart antennas

AI is known as one of the advanced computational techniques applied to various avenues of engineering and science. Industry 4.0 is no exception in this regard, and applications of AI in smart factories and modern industries are at their full potential [109–115]. In recent years, AI and ML algorithms have been applied to electromagnetic problems and antennas [71], paving the way toward true intelligent antenna systems with real-time agility.

In view of the aforementioned 2 smart antenna types, the implementation of the quick mapping from the coding patterns (i.e., states of the active components such as PIN diodes or varactor diodes) to the radiation patterns, and vice versa, for electrically large metasurfaces with complicated topologies is challenging. However, this becomes easy and realizable using AI/ML-based algorithms. As such, a pioneer work based on a physics-inspired neural network with few-shot learning ability is proposed for intelligent antenna beamforming in Ref. [71]. The average code prediction accuracy was greater than 98.4%. Physics-inspired neural network and deep neural network algorithms were combined to create an intelligent beamforming method that could accurately determine the necessary coding sequence for the desired antenna radiation beams in real time. Such intelligent methods can be developed to dynamically adapt the excitation coefficient for each array antenna element in response to changes in the electromagnetic environment (such as changing industrial settings), surrounding an array antenna [116–118].

Furthermore, using support vector machines, the antenna array can be trained to dynamically change the phase or excitation distribution in the antenna elements to maintain a specific radiation pattern. This helps to enhance the beam-scanning as well as null-steering capabilities to solve the direction of arrival (DoA) problems [119–122]. However, the development of AI-based smart antennas is not fully grown and is still envisioned to provide full physical layer-based control for future applications in Industry 4.0 and beyond.

Table 2 provides a summary of various agile smart antennas (capable of radiation pattern, polarization, and/or frequency reconfigurability). Moreover, a summary of the advantages, drawbacks, and challenges of conventional reconfigurable antennas, DMAs, and AI/ML-based reconfigurable antennas is presented in Table 3.

**Table 2.** Summary of various reconfigurable/tunable antennas at sub-6-GHz bands.

Reference	Operating frequency band (GHz)	Antenna element type	Material used	Beam-steering/ Reconfigurability method	Array geometry	Antenna gain (dBi)	Smart reconfigure feature
[82]	4.9 to 5.9	Microstrip	F4B TM-2	SP4T and phase shifter	Linear and planar, 1 × 4, 2 × 4, and 4 × 4	11.16 to 17.25	Selectable sectoral coverage from 90° to 360°
[52]	1.35 to 2.19	Microstrip	Multilayer, RO 4350, and FR4	Varactor diode	Planar, 4 × 4	11.85 to 15.28	Polarization agility and frequency reconfigurability
[32]	9.6 to 10.2	Microstrip	Multilayer, RO 4350B	PIN diode	Linear, 1 × 32	17.8	Beam-scanning from −60° to 60°
[86]	5.1 to 5.9	Microstrip	Multilayer, R05870, and RO5880	PIN diode	Circular configuration	10	360° beam-steering
[51]	1.5 to 2.4	Microstrip	Rogers 5880	Varactor diode	Circular, 4 elements	13.29	Frequency agility and polarization reconfigurability
[172]	1.97 to 2.54	Microstrip	FR4	Varactor	Triangular	3.1	Frequency, polarization and pattern reconfiguration
[173]	4.63 to 5.07	Microstrip	Multilayer, RO4350, RO4450, TLY-5	Pin diodes and RF switch	Planar array	7.5 to 10.5	Beam switching and polarization reconfiguration
[174]	2.4 to 2.48	Microstrip	RO 5880	Butler matrix	Linear 1 × 4	11.04	Beamforming using Butler matrix
[175]	0.9 to 1.5	Microstrip	Multilayer, RO4003C, 4450B	RF MEMS switch	Single element	4.5 to 6.5	Frequency agility and 4-state polarization diversity
[176]	1 to 3	Microstrip	Rogers 5880	PIN diode	Circular configuration	−3 to 3.2	Frequency and polarization reconfigurable
[81]	2.4	Microstrip	FR4	PIN diode	Single element	5.18 to 5.32	Beam-steering in 4 directions
[177]	2.4	Microstrip	FR4	PIN diode	Circular configuration	4.5	Pattern reconfigurability
[178]	1.8 to 2.2, 2.85 to 3.15	Monopoles and parasitic elements	-	PIN diode	Circular configuration	5.5	360° beam-steering and adaptive beamforming
[179]	2.3 to 2.55	Folded monopoles with parasitic elements	FR4	Varactor	Circular configuration	4	Beamforming and 360° beam-steering in azimuth
[180]	2.4 to 2.5	Microstrip	RO 4003C	PIN diode	Single element	6.5	Beam-steering
[181]	2.4	Microstrip	Taconic (TLY-5)	PIN diode	Circular configuration	7.01 to 8.01	Multidirectional pattern reconfiguration
[182]	3.44 to 3.6	Microstrip	-	PIN diode	Circular configuration	8	Pattern reconfiguration
[183]	5 to 5.2	Yagi-Uda dipole	R0 6010	PIN diode	Planar	7.5 to 10	Beam switching and pattern reconfiguration
[88]	2.36 to 2.6	Metamaterial	RO 4003C	Varactor	Planar	5.77 to 6.05	Pattern reconfigurability
[90]	10 GHz	Metamaterial	Multilayer, RO 4003C	Varactor	Planar	5 to 10	Beam-steering

**Table 3.** Summary of the advantages, prospects, and drawbacks of conventional reconfigurable antennas, DMAs, and AI/ML-based smart antennas.

Smart antenna type	Design philosophy	Advantages/Prospects	Drawbacks/Challenges
Dynamic reconfigurable antennas using conventional antenna elements	Based on an array of antenna elements, each of which serves as a radiator	Relatively easier to design and model	Relatively large size as compared to DMA with the same physical area
	Each antenna element is given active RF feed through a power divider (or an active feed network)	Feed is provided to each radiating antenna element; therefore, radiation efficiency is usually high	Design of efficient power division is difficult and usually lossy
	Each antenna element is always radiating, as it gets direct feed. Switching the state of the PIN diode simply changes the phase in each element	Availability of different types of feed networks, such as corporate feed, series feed, or corporate-series feed network	Phase arrays with bulky phase shifters consume more power and are costly
		Well-established literature and experimental verifications	
Dynamic metasurface antennas (DMAs)	Based on the waveguide structure, such as the microstrip waveguide structure	Small size of meta-element as compared to the conventional antenna element	Hardware design challenges to accurately model waveguide structure for electromagnetic coupling of the wave with meta-elements
	Basic radiator is a meta-element, which is excited when it is coupled to the waveguide structure	No need for active phase shifters, or complicated corporate feed network	Difficult to provide sufficient coupling of signal to each meta-element, thus needs careful waveguide design considerations
	Switching ON the PIN diode (forward bias state) results in loss of radiation characteristics of the meta-element. Switching OFF the PIN diode results in the radiation of the meta-element, as it couples with the waveguide structure	Low cost and low power as compared to dynamic conventional antenna arrays (e.g., those based on patch elements and phase shifters)	Challenging to handle insertion loss of meta-elements in the ON state when coupled with a waveguide aperture
		Lower number of RF chains required in case of digital beam processing with DMAs with enhanced performance, as compared to conventional dynamic antennas where the number of RF chains = the number of antenna elements. Therefore, a DMA manifests inherent so-called hybrid beamforming	Overall reduced radiation efficiency due to increased dielectric and conduction losses inside the waveguide structure as compared to that of conventional reconfigurable antennas
		Has the potential to be used at base station/access point as a multitude of radiating elements in massive MIMO communication even at sub-6-GHz bands	The system model and signal processing of multi-port DMA (or 2-D DMA) is complex
		Analysis of 1-D waveguide DMAs is easier with single mode as the wave propagates in one direction	Less established experimental research work
			Less established literature
AI/ML-based dynamic reconfigurable antennas	AI/ML antenna technique can be used with both types of antennas stated above, for real-time beam-switching	A trained AI-based model can steer the radiation pattern of antenna toward the required angular direction in real time	Challenging to train the AI model for a large number of possible antenna states and obtain accurate results. For instance, for 16 antenna elements/meta-elements, the possible coding combinations (or thus the number of radiation beams) will be 2^16^ = 65,536, and so on
		Very efficient for real-time dynamic reconfigurable antennas to achieve agility in industrial settings to ensure URLLC	Little established work in the literature on antenna radiation pattern reconfigurability using AI/ML

## Critical Smart Antenna Design Challenges

It is always important to undertake some important design factors while designing an antenna, for instance, antenna element characteristics, antenna array configuration, choice of substrate, conductor and dielectric losses, feeding mechanism, weight, cost, size, and easy integration with other RF circuitry.

After the right choice of the operating frequency band, the design of the radiating antenna element is the first step in any antenna design. Accurate modeling of the radiating element to achieve the desired bandwidth, radiation efficiency, polarization, and radiation pattern is crucial. The geometry of the antenna element, type of feed selection, and effect of the ground plane are some of the key factors to achieve optimum antenna performance.

In case of array design, the mutual coupling and a suitable distance between the antenna elements pose additional challenges [21,123–127]. It is important to control spurious radiations from feeding and power divider networks in array design. The mutual coupling between array elements is also a serious concern in array antennas. Different mutual coupling reduction techniques are proposed in the literature to achieve decoupling effects. Some of these techniques include electromagnetic bandgap structures, split ring resonators, metamaterial-based slabs, and defected ground structures [128–136]. The presence of the side lobes (grating lobes) in array antennas is another serious problem, which is the main cause of waste of energy in an undesired direction, reduction in the aperture efficiency, and the saturation of the radio receiver. Thus, the overall performance of the antenna system is degraded especially in a harsh industrial environment. Different smart techniques are proposed in the literature to reduce the grating lobes [137–142].

From an industrial point of view, it is found that the choice of antenna substrate material should be robust and durable to withstand harsh industrial environmental conditions, such as vibrations and temperature [143]. Some of these substrate choices include Arlon and polyvinyl chloride [143–145]. FR4 is fire-resistant and robust, often employed at low-frequency bands, but suffers from high losses. For higher-frequency bands, Rogers is a low-loss choice. Moreover, flexible substrates may also be used to design conformal antennas.

Accurate phase control using PIN diodes or varactor diodes with proper biasing is a challenging task in dynamic tunable antennas. Separation of RF source from DC (direct current) biasing source needs special care while designing reconfigurable antennas. Usually, radial stubs (act as AC by-pass capacitors) and thin transmission lines (as DC coupling inductors) are designed in an appropriate configuration to avoid DC offset, such as depicted in Refs. [78,103]. Designing an integrated phase shifting mechanism (using varactor/PIN diodes) on the same antenna aperture while preserving high aperture efficiency is difficult and needs extensive optimization. Furthermore, the efficient design of DMAs is a challenging task especially at high frequencies, as this is a relatively new research direction for a new class of smart antennas with less established experimental work and prototypes.

Other challenges in high-mobility scenarios include adaptive antenna beamforming, which deals with a unique challenge where the DoA changes quickly. It degrades the beamforming performance or even fails if not tackled properly [142]. Different challenges related to beamformer designs at sub-6 GHz as well as higher-frequency bands are highlighted in Ref. [146]. Apart from efficient and smart antenna performance, the other challenges are to develop efficient dynamic beamforming algorithms, and digital processing hardware to name but a few [146].

Finally, antenna characterization and measurements in a realistic harsh industrial environment is a challenging task. This is due to the strong reflective nature of the industrial environment and dynamic blockages. Moreover, wireless channel modeling of a realistic industrial environment is essential to provide precise specifications for required smart antennas.

## Conclusion and Future Perspectives

An inclusive survey on intelligent beam-steerable antenna systems from the viewpoint of Industry 4.0 applications is presented. The focus of smart antenna design was laid on sub-6-GHz ISM bands including Wi-Fi 6/6E and Wi-Fi 7, along with an overview of the industrial revolution process, wireless communication infrastructure, and key performance requirements at the physical layer. An overview of the specific categorization of industrial activities, such as PA and FA along with their KIPs is presented. Then, from the antenna design perspective, the existing industrial wireless communication standards and potential frequency bands are reviewed. It is explored that since conventional sub-6-GHz ISM bands (2.4 and 5 GHz) are crowded, bandwidth, interference, spectral congestion, and jamming are critical issues that become major impediments to meet Industry 4.0 requirements. Consequently, Wi-Fi 6/6E and Wi-Fi 7 standards are highlighted to play a key role to boost the efficiency and throughput of smart industrial applications, such as Industrial IoT and smart manufacturing.

From the perspective of Industry 4.0 and beyond, dynamic beam-steerable antennas are crucial to provide real-time and seamless wireless connectivity for changing manufacturing and factory line conditions. In this view, various state-of-the-art sub-6-GHz smart antenna designs are explored and presented in this work. It was analyzed that adaptive beam patterns, dynamic beamforming, and beam steerability, as well as frequency, polarization, and radiation pattern agility, are the key features to achieve efficient communication for Industry 4.0 and beyond at sub-6-GHz ISM bands. Reconfigurable antennas (using conventional elements), DMAs with beam-steering capabilities, and AI-based dynamic reconfigurable antennas are reviewed and indicated as key enablers for modern WLAN/WPAN industrial communication as well as Industrial IoT.

Different challenges and design considerations involved in the smart antenna design process are elucidated in this work. In addition, it is inferred that reconfigurability and agility in frequency, polarization, and especially in radiation pattern are necessary to design a robust smart antenna to mitigate congested sub-6-GHz ISM band industrial communication. It is proposed that Wi-Fi 6- and Wi-Fi 7-based antenna designs integrated with adaptive beamforming algorithms and smart architecture can efficiently fulfill URLLC and high-throughput demands with seamless connectivity in industrial settings.

**Fig. 10.**
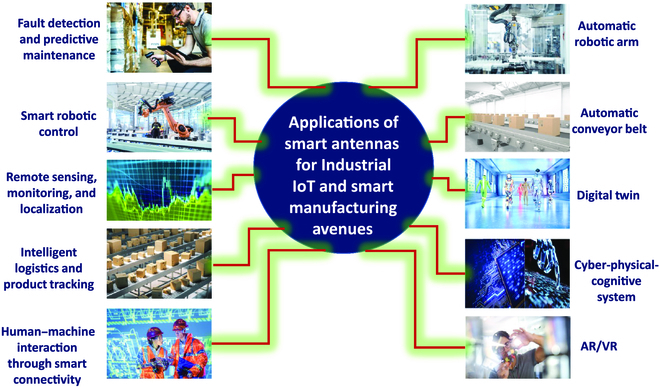
An outlook of the future applications of smart antennas in Industry 4.0 and beyond avenues.

Before we conclude this review, it is important to unveil some important future directions in this field. Industrial IoT is the basic pillar of digital manufacturing and comprises many smart sensors, equipment, tools, software platforms, and cloud servers. The objective is to connect all industrial assets, including machines and control systems, with information systems and business processes. For specialized applications, smart sensors are used at all stages of the smart manufacturing process. The IoT gateway, which serves as a hub for connecting Industrial IoT devices and the cloud, receives and transmits wireless data from these sensor networks through smart antennas constantly to the cloud application server for processing and analysis. As a result, the vast amount of data gathered can support analytical approaches and result in effective industrial operations. The purpose of smart manufacturing, on the other hand, is to promptly and dynamically adapt to changes in demand during the production phase of a smart product life cycle. Consequently, the Industrial IoT has an impact on the whole industrial value chain and is necessary for smart production. Since an antenna is an integral part of the Industrial IoT system, it is necessary to put immense consideration on smart antenna designs and employ them as potential PHY solutions, so that Industry 4.0 and beyond could soar to full potential. An outlook of potential applications of smart antennas in Industrial IoT and related use cases of smart manufacturing is presented in Fig. 10.

Furthermore, we highlight massive MIMO, Intelligent Reflecting Surface (IRS)-based industrial communication, and 6G technology as some of the new PHY frontiers that have amazing prospects and potential for remarkable next-generation industrial applications.

### Massive MIMO and multiple antenna technology

Multiple antenna technology has made its way into mainstream communication systems and attained immense research interest. Spatial multiplexing and adaptive beamforming are the main benefits of multiple antenna technology, which lead to high data rate, reliability, and traffic demands [147,148]. MIMO is a multiple antenna technology that refers to the use of multiple antennas at the base station (transmitter side) through which the spectral, spatial, and energy efficiency can be improved by orders of magnitude [149].

The concept of massive MIMO has taken immense value in research to meet 5G and 6G technological requirements as presented in Ref. [50] and Refs. [150–154]. Fully digital receivers for massive MIMO have been developed and tested commercially at sub-6-GHz bands [155,156]. Since the spectral efficiency between the transmitter antenna and the receiver antenna in a typical industrial environment is restricted by the channel’s attenuation, and interference from other transmissions at the same time and frequency resources, massive MIMO can be a sophisticated choice to enhance the spectral efficiency and signal power to manifold. The inspiration can be sought from recent works on massive MIMO [151–154].

### Intelligent reflecting surface

IRS is a revolutionary technique for reconfiguring the wireless propagation environment through software-controlled reflections to achieve enhanced spectral and energy efficiency in a cost-effective manner [157–160]. IRS is also known under other names as Reconfigurable Intelligent Surface or Software-Controlled Metasurface. The elements of an IRS can independently reflect the incident signal by controlling the phase and/or amplitude. In this way, passive beamforming is collaboratively achieved for directional signal enhancement or null placement. Practically, the development of an IRS includes reflective elements, phase-switching components such as PIN diodes or varactors, and external control circuitry [161]. Further details about the design considerations of the IRS can be found in Refs. [162–164], to list but a few.

Here, we tend to highlight the promising potential of the IRS technique to boost industrial communication performance and control in the future. Wireless signals are easily blocked by big structures in industrial automation due to densely distributed equipment such as metal machinery, erratic movement of items (robots and trucks), wooden structures, and thick pillars. Consequently, performance reliability is degraded. In this scenario, the IRS is a promising candidate to provide an alternate transmission link when the direct link is obstructed [11,165]. The communication link can be established through an IRS installed on the ceiling or wall of the smart factory, thus providing seamless reliable connectivity for mission-critical industrial communication [166]. Since the practical deployment of IRS technology in industrial settings is still in its infancy, it is an inspiring area for research to explore challenges and opportunities. A vision to make IRS-enabled smart industries seems to be realized in the near future.

### 6G technology (mmWave and THz communication)

The 6G technology is anticipated to be embraced by smart industries. It aims to fully integrate advanced features, such as AI, autonomous vehicles, mixed reality, and haptic interface technologies that require extremely low latency communication and are essential features for Industry 4.0 and beyond [167–169]. The 6G wireless systems will need greater improvements in terms of throughput (up to 1 Tbps), network capacity (1,000× 5G capacity), energy efficiency, backhaul and access network congestion, and data security in order to achieve this integration. Millimeter-wave and terahertz (THz) communication [18,159 ,170 ,171], optical wireless communication [31], and dynamically controllable wireless systems [32,50,70,71,159 ,171] are some of the key enablers to envision 6G for future industrial applications.

As a concluding remark, we discussed important issues and revealed possible directions for future wireless industrial applications. The potential of Wi-Fi 6 and Wi-Fi 7 as efficient sub-6-GHz bands for wireless industrial communication is unfolded. An extensive insight is presented on the potential of smart dynamic reconfigurable antennas as a PHY layer solution for next-generation industrial applications such as Industrial IoT and flexible manufacturing. It is expected that this paper will serve as a catalyst and an inspiring resource to motivate further research in this area.

## Data Availability

The data could be given upon reasonable request from the corresponding authors.
